# Effect of hydro-alcoholic extract of *Cinnamomum zeylanicum *on nitric oxide metabolites in brain tissues following seizures induced by pentylenetetrazole in mice 

**DOI:** 10.22038/AJP.2022.19578

**Published:** 2022

**Authors:** Amir Hossein Assaran, Farimah Beheshti, Narges Marefati, Roghayeh Rashidi, Mahmoud Hosseini, Bahram Bibak, Farzaneh Shakeri

**Affiliations:** 1 *Applied Biomedical Research Center, Mashhad University of Medical Sciences, Mashhad, Iran*; 2 *Neuroscience Research Center, Torbat Heydariyeh University of Medical Sciences, Torbat Heydariyeh, Iran*; 3 *Department of Physiology, School of Paramedical Sciences, Torbat Heydariyeh University of Medical Sciences, Torbat Heydariyeh, Iran *; 4 *Department of Physiology and Medical Physics, Faculty of Medicine, Baqiyatallah University of Medical Sciences, Tehran, Iran*; 5 *Pharmacological Research Center of Medicinal Plants, Mashhad University of Medical Sciences, Mashhad, Iran*; 6 *Psychiatry and Behavioral Sciences Research Center, Mashhad University of Medical Sciences, Mashhad, Iran*; 7 *Neuroscience Research Center, Mashhad University of Medical Sciences, Mashhad, Iran*; 8 *Natural Products and Medicinal Plants Research Center, North Khorasan University of Medical Sciences, Bojnurd, Iran*; 9 *Department of Physiology and Pharmacology, School of Medicine, North Khorasan University of Medical Sciences, Bojnurd, Iran*

**Keywords:** Pentylenetetrazole, Seizures, Mice, Nitric oxide, Oxidative stress, Brain

## Abstract

**Objective:**

The effects of *Cinnamomum zeylanicum* on oxidative stress imposed by pentylenetetrazole (PTZ) was examined in mice brain tissues.

**Materials and Methods:**

Animals were divided into five groups as follows: 1- control group which received saline; 2- PTZ group (100 mg/kg, ip); and groups 3 to 5 which received (100, 200, and 400 mg/kg) of *C. zeylanicum* for seven days prior to PTZ injection. The latencies of the first minimal clonic seizure (MCS) and the first generalized tonic-clonic seizure (GTCS) and levels of oxidant and antioxidant biomarkers were measured.

**Results:**

Treatment with the two higher doses of the extract significantly increased the MCS and GTCS latencies (p<0.05 to p<0.001). Malondialdehyde (MDA) and nitric oxide (NO) levels were increased, but superoxide dismutase (SOD), catalase (CAT), and thiol were decreased in both cortical and hippocampal tissues of the PTZ group compared to the controls (p<0.001). Pretreatment with the two higher doses of *C. zeylanicum* significantly led to a significant correction in NO, MDA, SOD and CAT levels in the hippocampus and cortex compared to the PTZ group (p<0.05 to p<0.001).

**Conclusion:**

Antioxidant and anticonvulsant effects of *C. zeylanicum* in PTZ-injected animals may suggest its potential therapeutic effect on nervous diseases such as seizures.

## Introduction

A seizure attack is generally characterized by behavioral changes and physical features which are followed by an episode of abnormal brain electrical activity and although it is a main feature of epilepsy disease, it also occurs in other conditions including hypoglycemia, hypocalcaemia and fever (Fisher et al., 2014 ▶). Epilepsy is a central nervous system (CNS) disorder in which, brain function becomes irregular, causing seizures or periods of odd behavior, sensations, and often loss of consciousness (Trinka et al., 2015 ▶). Epilepsy decreases the quality of life and raises the risk of disability and even death (Jalili et al., 2014 ▶). 

Nitric oxide (NO) is produced by NO synthases, which transform L-arginine to L-citrulline. NO is a critical component of blood flow control in the brain. As an excitatory neurotransmitter that participates in synaptic plasticity, it also influences complex neural functions such as brain development, memory formation, and behavior. Overproduction of NO, on the other hand, has been linked to neurotoxicity in ischemia, certain types of neurodegenerative brain diseases, and seizure induction (Garthwaite, 1991 ▶). NO has a complex effect on experimentally-induced seizures. NO has several other effects in addition to cyclic guanosine monophosphate (cGMP) stimulation including blocking N-Methyl-D-aspartic acid or N-Methyl-D-aspartate (NMDA) receptors in a negative manner, thereby reducing the excitability of the receptor (Manzoni et al., 1992 ▶) , glutamate release (Pelligrino et al., 1996 ▶) and decreasing the inhibitory activity of gamma aminobutyric acid (GABA) neurotransmitter receptor (Robello et al., 1996 ▶). 

The induction of seizures by pentylenetetrazole (PTZ) may be linked to its antagonistic activity on GABA-A receptor as well as NMDA-receptor activation (Kaputlu and Uzbay, 1997 ▶). As a result, the induced NO can increase PTZ ability to cause convulsions in both directions. Overproduction of NO has also been linked to oxidative damage in brain tissues (Picón-Pagès et al., 2019 ▶). Oxidative stress has been identified as a mismatch between generation and removal of reactive oxygen species (ROS), (Sahebari et al., 2015 ▶). Oxidative stress is thought to be a potential cause for epilepsy pathogenesis (Chang and Yu, 2010 ▶). Studies have also confirmed that some of the epilepsy symptoms could be due to oxidative stress-related brain damage (Mehla et al., 2010 ▶).

Benzodiazepines, barbiturates, GABA analogues, succinimides, hydantoins, and carbamazepine are some of the medications commonly used to treat epilepsy (Goldenberg, 2010 ▶). There are challenges in the treatment of different forms of epilepsy, despite recent advances in antiepileptic medications (Sendrowski and Sobaniec, 2013 ▶). In addition, about 30% of patients are known to have pharmaco-resistant epilepsy and may not react to these drugs, and there are some problems with the treatment process, such as severe side effects and chronic drug toxicity (Hitiris et al., 2007 ▶). There is also a growing interest in the use of plants and other natural resources for the development of new therapeutic drugs.

*Cinnamomum zeylanicum*, also known as Ceylon cinnamon belonging to Lauraceae family, is indigenous to Sri Lanka, Indochina, and Madagascar and India. *C. zeylanicum* inner bark has been used as a powerful therapeutic agent as well as a flavoring ingredient in foods (Unlu et al., 2010 ▶). Different pharmacological properties such as anti-proliferative (Alizadeh Behbahani et al., 2020 ▶) and anti-inflammatory (Gunawardena et al., 2015 ▶) effects for this plant have been reported. Antioxidant effect of *C. zeylanicum* was also demonstrated due to its high amount of phenolic compounds (Ghosh et al., 2015 ▶). 

In this study, the effects of *C. zeylanicum* hydroethanolic extract on the latencies of the first minimal clonic seizure (MCS) and the first generalized tonic-clonic seizure (GTCS), as well as the levels of NO, malondialdehyde (MDA), catalase (CAT), superoxide dismutase (SOD), and thiol in the hippocampus and cortex tissues after seizures induced by PTZ in mice were investigated.

## Materials and Methods

**Animals**

Male mice (20-30 g) were purchased from the animal house of North Khorasan University of Medical Sciences and kept in a controlled room (21-22°C) with a 12-hr light/12-hr dark cycle. The North Khorasan University of Medical Sciences Ethics Committee approved the study (Ethics allowance No. 970048).

**Plant extraction**

The *C. zeylanicum* barks were purchased from Bojnurd, North Khorasan province, Iran. The barks of *C. zeylanicum* were grounded to powder and extracted with ethanol (70%) in a Soxhlet extractor. The resultant extract was concentrated under low pressure. The solution was then dried using a water bath. The dried extract was maintained at -20°C until it was used. Saline was used to dissolve the extract before administration. 

**Experimental groups**

**Figure 1:**
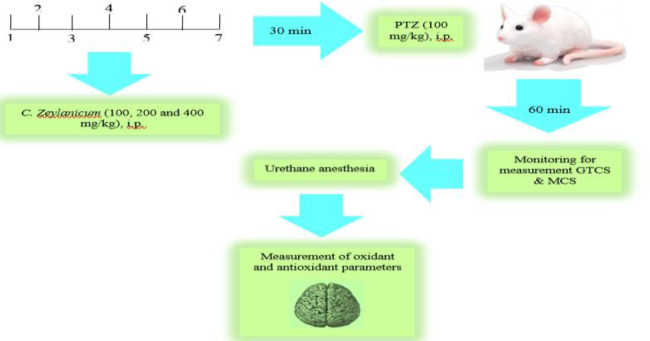
Schematic description of study design

Animals were randomly divided into five groups (n=8 in each group) including: 1) control group (saline), 2) PTZ group (100 mg/kg, i.p.), and 3-5) groups including PTZ-Extract 100 mg/kg (PTZ-Ext 100), PTZ-Extract 200 mg/kg (PTZ-Ext 200) and PTZ-Extract 400 mg/kg (PTZ-Ext 400) that received *C. zeylanicum* hydroethanolic extracts at three doses of 100, 200 and 400 mg/kg, i.p. once daily for one week before PTZ injection (Seema and Sparsh, 2019 ▶), (Figure 1). The extract in these doses are not toxic (Shah et al., 1998 ▶).

**Induction of seizures by PTZ**

PTZ, is a selective inhibitor of the chloride channel and is an antagonist for GABA receptor. It is a well-known chemoconvulsant which is frequently used for evaluation of antiepileptic drugs. PTZ at high doses produces a continued seizure activity which progresses from mild myoclonic jerks to face and forelimbs clonus without loss of righting reflex (which is known as MCS), to clonic seizures of limbs with loss of righting reflex which is followed by full tonic extension of both forelimbs and hind limbs (GTCS). PTZ has been repeatedly used at 90-100 mg/kg to induce MCS and GTCS seizures (Karami et al., 2015 ▶; Anaeigoudari et al., 2016 ▶; Choopankareh et al., 2015 ▶).

 In the current study, 100 mg/kg of PTZ was injected i.p. to induce a seizure experimental model. PTZ injection was done 30 min after the last saline administration or different hydroethanolic extract of *C. Zeylanicum* (100, 200 and 400 mg/kg). The mice were then mounted in a Plexiglass box (30×30×30 cm) were monitored for 60 min after the PTZ injection (Figure 1). Then, the latency of the first generalized tonic–clonic seizures (GTCS) and latency of first minimal clonic seizure (MCS) were measured (Khodabakhshi et al., 2017 ▶). 

**Measurement of oxidant and antioxidant levels**

Following behavioral testing, the mice were rapidly decapitated, their brains were removed, and the cortical and hippocampal regions were dissected on an ice-cold surface and homogenized in ice-cold phosphate-buffered saline to achieve 10% homogeneity and used for measurements of NO metabolites (Eftekhar et al., 2019 ▶), and oxidant and antioxidant biomarkers (Khodabakhshi et al., 2017 ▶).

Concentration of NO metabolites was measured using the Griess reagent kit (Promega Co.).

The levels of malondialdehyde (MDA), as an index of lipid peroxidation, were measured. MDA reacts with thiobarbituric acid (TBA) as a thiobarbituric acid reactive substance (TBARS) to produce a red complex with the maximum absorbance at 535 nm. 

Total thiol concentration was measured using DTNB reagent which reacts with thiol moieties to produce a yellow complex with the maximum absorbance at 412 nm. Briefly, 1 ml tris-ethylene diamine tetra acetic acid (Tris-EDTA) buffer (pH 8.6) was added to 50 μl supernatant in 1-ml cuvettes and sample absorbance was read at 412 nm against Tris-EDTA buffer alone (A_1_). Then, 20 μl of DTNB reagents (10 mmol/l in methanol) was added to the mixture and after 15 min (at room temperature), the sample absorbance was read again (A_2_). The absorbance of DTNB reagent alone was also read as blank (B). Total thiol concentration (mmol/l) was calculated using the following equation: Total thiol concentration (mmol/l) = (A_2_–A_1_–B)×1.07/(0.05×13.6).

A colorimetric assay developed based on the production of superoxide through pyrogallol auto-oxidation and inhibition of superoxide-dependent diminution of the tetrazolium dye (MTT; (3-(4,5-dimethylthiazol-2-yl) 2,5-diphenyltetrazolium bromide)) to formazan by SOD, was used at 570 nm. One unit of SOD activity was defined as the quantity of enzyme required for 50% inhibition of MTT reduction rate.

 Catalase (CAT) activity was assessed based on the H_2_O_2_-decomposition rate constant, k (dimension: s^-1^). Reductions in absorbance at 240 nm per minute and the rate constant of the enzyme were determined. Activities were defined as k (rate constant) per liter.

**Statistical analysis**

 The data in this study is presented as mean±SEM. Normality of the data was checked using the Kolmogorov-Smirnov test. Statistical comparisons were made using one-way ANOVA with the Tukey-Kramer *post hoc* test. The differences were considered statistically significant if the p value was less than 0.05.

## Results

**The effect of** ***C. zeylanicum*** **on MCS and GTCS**

**Figure 2:**
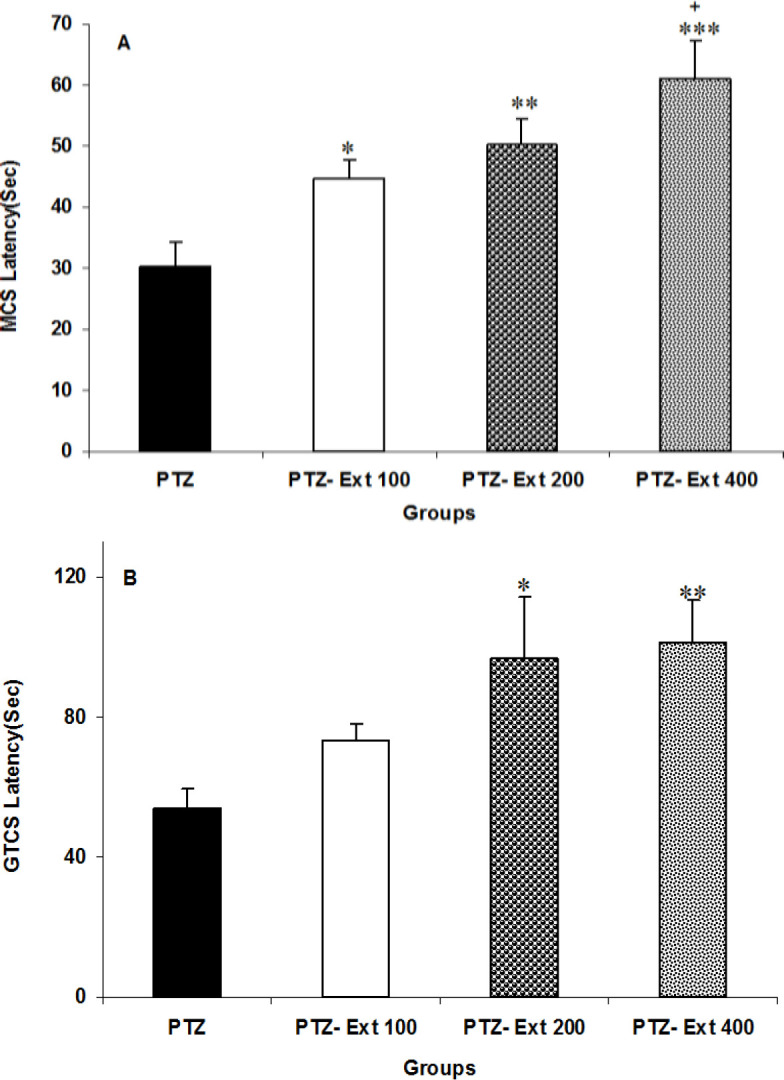
The effects of three doses (100, 200 and 400 mg/kg) of *C. zeylanicum* extract on the MCS (minimal clonic seizures) (A) and GTCS (generalized tonic–clonic seizures) (B) latencies. ^*^p<0.05, ^**^p<0.01 and ^***^p<0.001 as compared to the PTZ group, and^ +^p<0.05 as compared to the PTZ-Ext 100 group

Administration of three doses of *C. zeylanicum* significantly increased MCS latency compared to the PTZ group (p<0.05 to p<0.001). The effect of the highest dose of *C. zeylanicum* (400 mg/kg) on MCS latency significantly was higher than its lowest dose (100 mg/kg) (p<0.05, Figure 1A). Pretreatment with *C. zeylanicum* at medium and high doses (200 and 400 mg/kg) significantly delayed GTCS onsets (p<0.05 to p<0.01), whereas the low dose (100 mg/kg) had no effect on GTCS latency. There was no significant difference in GTCS latency among the three doses of the extract (Figure 2B). 

**The effect of** ***C. zeylanicum*** **on NO metabolites in the brain**

**Figure 3:**
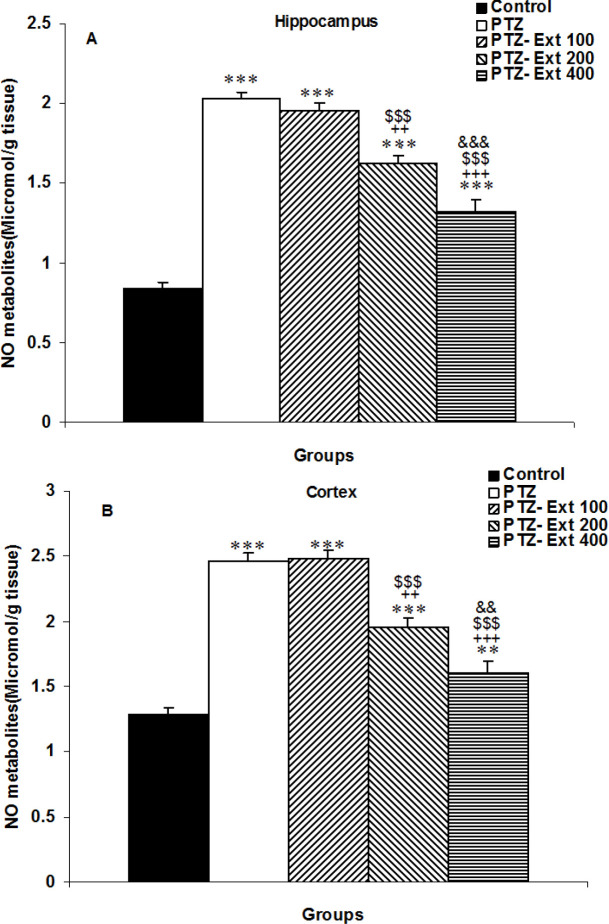
Comparison of the nitric oxide (NO) metabolites level in the hippocampus (A) and cortex (B) among the groups. ^**^p<0.01 and ^***^p<0.001 as compared to the control group,^ +++^p<0.001 as compared to the PTZ group, ^$$$^p<0.001 as compared to the PTZ-Ext 100 group, and ^&&^p<0.01 and ^&&&^p<0.001 as compared to the PTZ - Ext 200 group

The level of NO metabolites in the hippocampus and cortex of PTZ-injected animals was significantly higher than the control group (p<0.001 for both; Figure 3). The level of NO in the groups treated with two higher doses of *C. zeylanicum*, was significantly reduced compared to the PTZ group (p<0.001 in all cases); however, the lowest dose was not effective (Figure 3). Furthermore, NO metabolites in both the hippocampus and cortex of groups treated with 200 and 400 mg/ kg of the extract were significantly lower than the group treated with the lowest dose of the extract (p<0.001 in all cases). In addition, the level of NO in both the hippocampus and cortex of the animals treated with the highest dose of extract was lower than that in those treated by medium dose of the extract (p<0.001 for the hippocampus and p<0.01 for the cortex). Furthermore, the extract did not completely correct the level of NO metabolites, and NO metabolites in both the hippocampus and cortex of the groups treated with all doses of the extract were higher than the control group (p<0.01-p<0.001).

**The effect of** ***C. zeylanicum*** **on lipid peroxidation in the brain**

**Figure 4:**
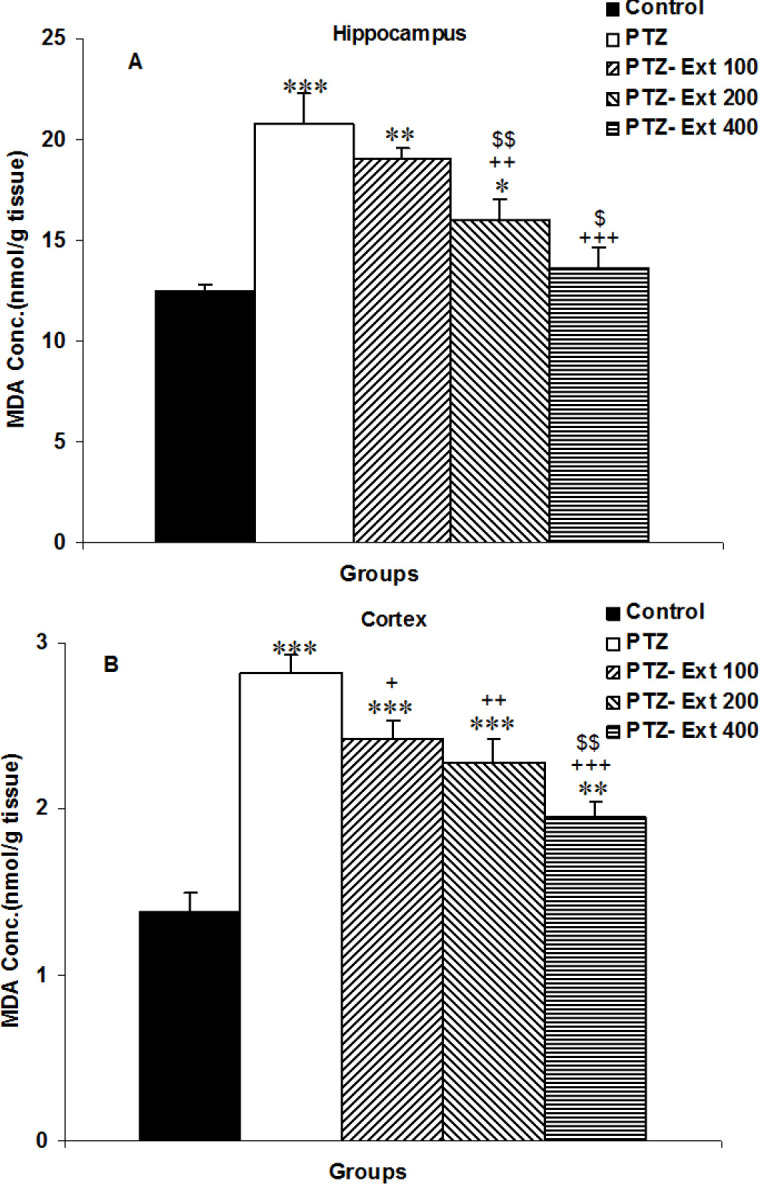
Comparison of the malondialdehyde (MDA) concentration in the hippocampus (A) and cortex (B) among the groups. ^*^p<0.05,^ **^p<0.01 and ^***^p<0.001 as compared to the control group,^ +^p<0.05,^ ++^p<0.01 and ^+++^p<0.001 as compared to the PTZ group, and ^$^p<0.05 and ^$$^p<0.01 as compared to the PTZ-Ext 100 group

In the hippocampus and cortex of PTZ-treated mice, the MDA level was considerably elevated relative to the control group (p<0.001 for both). The levels of MDA in the hippocampus of the groups treated with the two higher doses of the extract and in the cortex of those treated with all three doses of *C. zeylanicum *extract were lower than the PTZ-injected mice (p<0.01- p<0.001) however, the lowest dose of the extract was not effective to attenuate MDA in the hippocampus of the PTZ-Ext 100 group compared to the PTZ group. In addition, MDA level in the hippocampus of the rats treated with 200 and 400 mg/kg of the extract (p<0.01 and p<0.05, respectively) and in the cortex of the rats treated by 400 mg/kg of the extract (p<0.01) was lower than those treated with 100 mg/kg of the extract (Figure 4). Additionally, MDA level in the hippocampus of both the PTZ- Ext 100 and PTZ-Ext 200 groups and in the cortex of PTZ- Ext 100, PTZ-Ext 200 and PTZ-Ext 400 groups was higher than the control group (p<0.05- p<0.001); however, there was no significant difference in hippocampal MDA levels between the PTZ-Ext 400 and control groups (Figure 4). 

**The effect of** ***C. zeylanicum*** **on thiol content in the brain**

**Figure 5:**
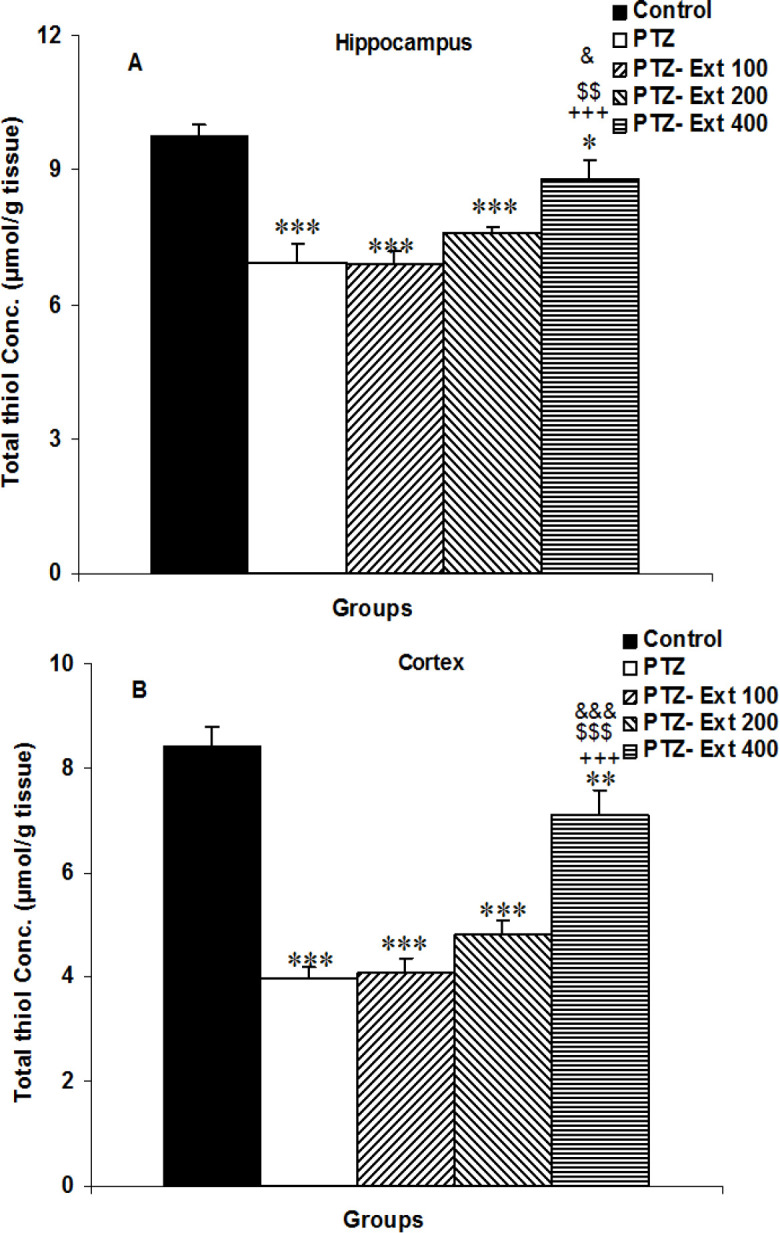
Comparison of the thiol concentration in the hippocampus (A) and cortex (B) among the groups. ^*^p<0.05,^ **^p<0.01 and ^***^p<0.001 as compared to the control group,^ +++^p<0.001 as compared to the PTZ group, ^$$$^p<0.001 as compared to the PTZ-Ext 100 group, and^ &^p<0.05 and ^&&&^p<0.001 as compared to the PTZ-Ext 200 group

Thiol level was reduced in the hippocampus and cortex of the PTZ animals compared to the control group (p<0.001 for both). The thiol levels in the hippocampus and cortex of the groups treated by 400 mg/ kg of the extract were higher than the PTZ group (p<0.001 for both); however, none of 100 and 200 mg/kg of the extract was effective (Figure 5). Both hippocampal and cortical thiol content in the PTZ-Ext 400 group was higher than those in the PTZ-Ext 100 and PTZ-Ext 200 groups (p<0.05 to p<0.001). Both hippocampal and cortical thiol level in PTZ- Ext 100, PTZ-Ext 200 and PTZ-Ext 400 groups was higher than the control group (p<0.05- p<0.001).

**The effect of** ***C. zeylanicum*** **on SOD activity in the brain**

**Figure 6:**
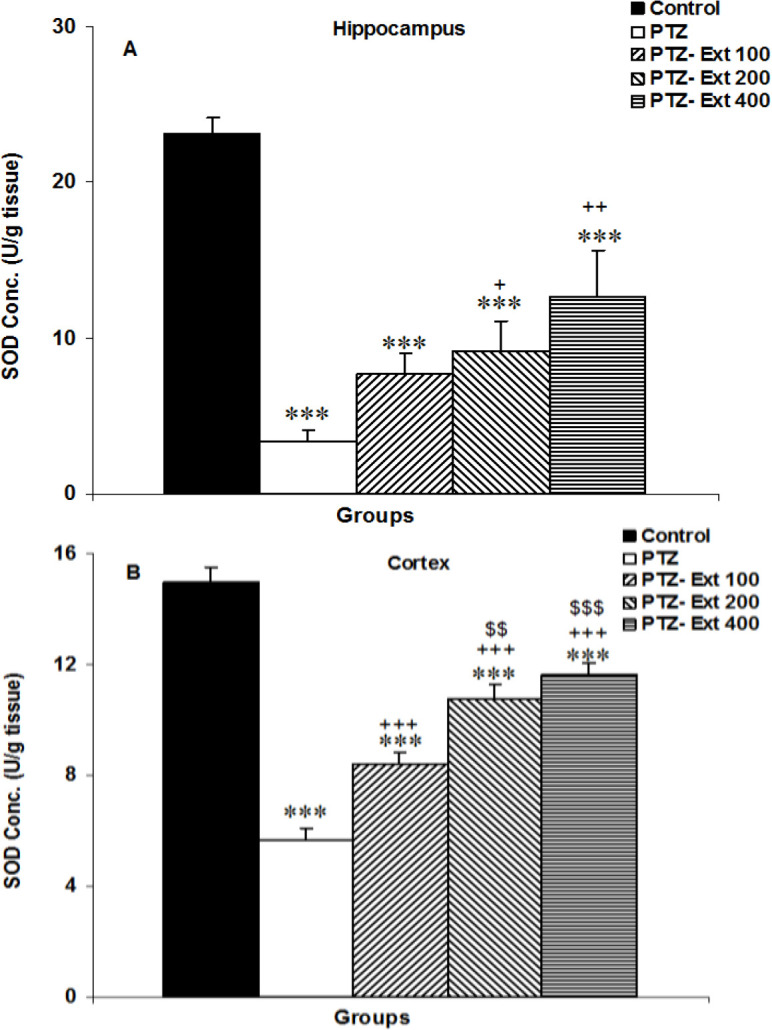
Comparison of the superoxide dismutase (SOD) activity in the hippocampus (A) and cortex (B) among the groups. ^***^p<0.001 as compared to the control group,^ +^p<0.05,^ ++^p<0.01 and^ +++^p<0.001 as compared to the PTZ group, ^$$^p<0.01 and ^$$$^p<0.001 as compared to the PTZ-Ext 100 group.

Seizure attacks induced by PTZ were followed by a decrease in SOD activity in the hippocampus and cortex (p<0.001 for both). Hippocampal activity in both PTZ-Ext 200 and PTZ-Ext 400 groups and cortical SOD activity in PTZ-Ext 100, PTZ-Ext 200 and PTZ-Ext 400 groups was higher than that in the PTZ group (p<0.05- p<0.001). Between the PTZ-Ext 100 and PTZ groups, there was no significant difference in hippocampal SOD activity (Figure 6). Cortical SOD in both PTZ-Ext 200 and PTZ-Ext 400 groups was higher than that in the PTZ-Ext 100 (p<0.01 and p<0.001, respectively). There was no significant difference among the groups treated by three doses of the extract in hippocampal SOD (Figure 5). Cortical and hippocampal SOD activities in PTZ-Ext 100, PTZ-Ext 200 and PTZ-Ext 400 groups were lower than that in the control group (p<0.001 for all).

**The effect of** ***C. zeylanicum*** **on CAT activity in the brain**

**Figure 7:**
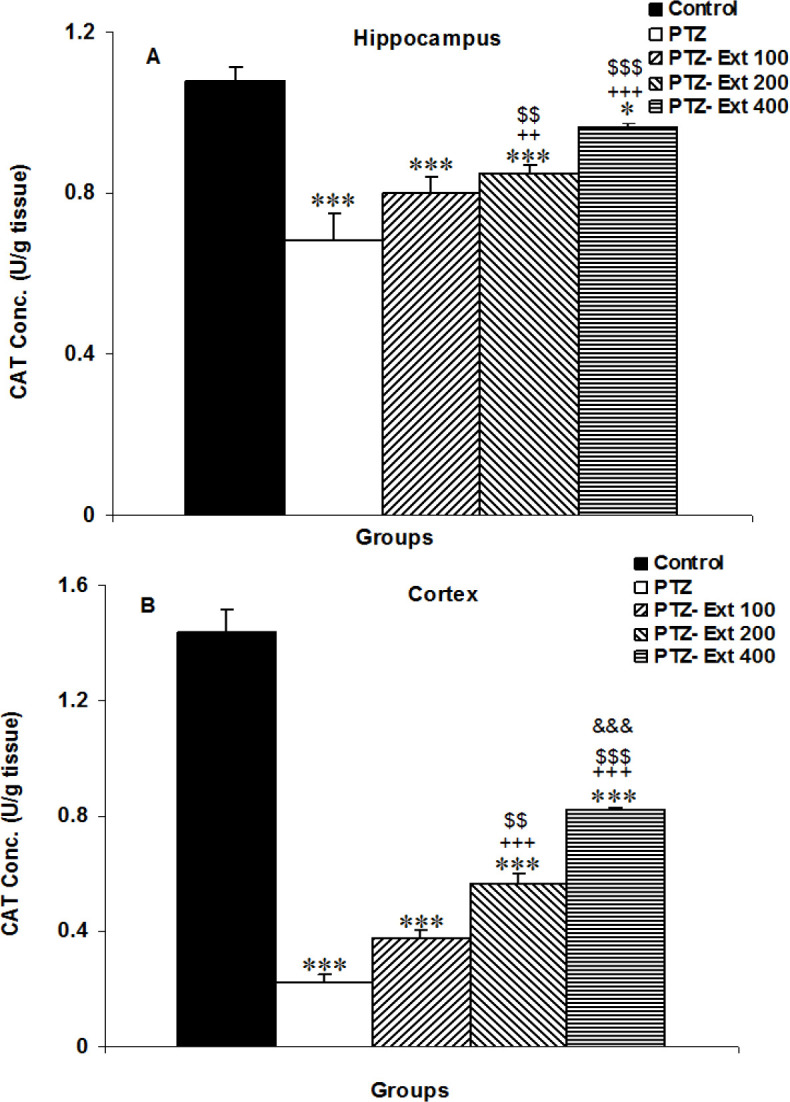
Comparison of the catalase (CAT) activity in the hippocampus (A) and cortex (B) among the groups. ^*^p<0.05 and ^***^p<0.001 as compared to the control group, ^++^p<0.01 and^ +++^p<0.001 as compared to the PTZ group, ^$$^p<0.01 and ^$$$^p<0.001 as compared to the PTZ-Ext 100 group, and^ &&&^p<0.001 as compared to the PTZ - Ext 200 group

Both hippocampal and cortical CAT in the PTZ group were lower than in the control group (p<0.001). Treatment by the medium and highest doses of the extract improved CAT activity in both the hippocampus and cortex of the PTZ-Ext 200 and PTZ-Ext 400 groups compared to the PTZ group (p<0.01-p<0.001); however the lowest dose was not effective (Figure 7). Hippocampal and cortical CAT activity in both the PTZ-Ext 200 and PTZ-Ext 400 groups was higher than the PTZ-Ext 100 group (p<0.01- p<0.001). In addition, cortical CAT activity in the PTZ-Ext 400 group was higher than the PTZ-Ext 200 group (p<0.001). Hippocampal and cortical CAT in PTZ-Ext 100, PTZ-Ext 200 and PTZ-Ext 400 groups did not reach the level of the control group (p<0.05- p<0.001). 

## Discussion

The results of the present study showed that *C. zeylanicum* extract postponed the seizure attacks in PTZ-induced animal model, attenuated NO metabolites and induced anti-oxidant effects.

PTZ is an antagonist of GABA-A receptor and it is a widely recognized chemical convulsant, often utilized in the evaluation of anti-epileptic drugs (Hosseinzadeh and Sadeghnia, 2007 ▶; Porter et al., 1984 ▶). A high dose of PTZ intraperitoneal injection causes a continuous seizure that ranges from mild myoclonic jerks to facial and forelimbs clonus without righting reflex loss (called MCS), to limb clonic seizures with righting reflex loss which is followed by complete tonic extension of both front and hind limbs (called GTCS), (Löscher et al., 1991 ▶). In the current research, PTZ injection was followed by MCS and GTCS seizures. Previous studies have also shown induction of MCS and GTCS in animals after PTZ injection (Homayoun et al., 2015 ▶; Khodabakhshi et al., 2017 ▶).

In this study, prior to PTZ injection, the experimental groups of mice received 100, 200, and 400 mg/kg of *C. zeylanicum* extract and MCS and GTCS were evaluated. The results showed that pretreatment with different doses of *C. zeylanicum* extract increased MCS and GTCS compared to the PTZ group. Our results are similar to a previous study in which, *C. zeylanicum* extract (250, 500, and 750 mg/kg, p.o.) showed anticonvulsant effects in maximal electroshock (MES) by tonic flexion and tonic extension and in PTZ model by delaying the onset time of seizures (Lodhi et al., 2019 ▶). 

Brain damage due to oxidative stress has been reported to occur as a consequence of seizures (Lin and Chen, 2020 ▶). Oxidative stress has also been suggested to have a role in the pathogenesis of epilepsy and seizures (Aguiar et al., 2012 ▶). Interestingly, NO has been considered to act as a free radical especially in an overproduced condition (Sahebari et al., 2015 ▶; Moncada and Bolaños, 2006 ▶). NO is also considered a trustworthy neuronal transmitter in the brain (Garthwaite et al., 1988 ▶). Depending on the type of seizure, it is either anticonvulsant or proconvulsant. In many *in vivo* and *in vitro* experiments, the role of NO in epilepsy has been investigated but the results are still inconsistent and both pro- and anti-convulsant properties of NO are recorded (Wojtal et al., 2003 ▶). For instance, NO plays an anticonvulsant role in bicuculline seizures or electrical seizures (Nidhi et al., 1999 ▶; Theard et al., 1995 ▶) while, it is a pro-convulsant one in PTZ-induced seizures (Riazi et al., 2006 ▶). PTZ via NMDA glutamate receptors activates calcium release that consequently activates calcium-calmodulin pathway to increase neuronal isoform of nitric-oxide synthase (nNOS) protein expression and NO level is able to increase the induction of generalized clonic-tonic seizures (Itoh and Watanabe, 2009 ▶). Considering the high amount of NO in the brain of the PTZ injected mice which was observed in the present study, it might be suggested that NO has a role in the seizure attacks induced by PTZ; however, more precise studies are needed to be done using electro cortical (ECOG) recordings. More cellular and molecular experiments are suggested to be done in the future.

On the other hand, high levels of NO in the brain of PTZ-injected animals were accompanied with a high level of MDA and a decrease of thiol, SOD and CAT. Considering these results, it seems that NO overproduction plays an important role both as a consequence and as a cause of epileptic seizures. Previous studies indicate that oxidative stress is important in damage to brain tissues after seizure induction (Kaneko et al., 2002 ▶; Kudin et al., 2002 ▶; Liang and Patel, 2004 ▶). Protein structure damage and lipid oxidation were recorded in the hippocampus 4 and 24 hr after seizure induction and it was associated with seizure activity (Kim et al., 1997 ▶). These differences may be due to some limitations of the present study, such as the number of animals and short-term period of treatment.

It is hypothesized that oxidative stress has a role in the pathogenesis of epilepsy (Devi et al., 2008 ▶). This theory can be supported by the presence of a high level of reactive oxygen species (ROS) in the brain, including superoxide anions, hydroxyl radicals and hydrogen peroxide following seizures (Devi et al., 2008 ▶). However, it has also been well established that oxidative damage to brain tissues plays a part in the pathogenesis of the symptoms of epilepsy (Kudin et al., 2002 ▶). An enhanced generation of products of lipid peroxidation and reduction of SOD, GSH and glutathione peroxidase (GPx) in cerebral tissues of rats with PTZ-induced seizures has been reported (Guna et al., 2018 ▶). 

The results of the present study also showed that pretreatment with various doses of *C. zeylanicum* extract increased SOD, CAT, and thiol levels but reduced levels of NO and MDA in the hippocampus and cortex tissues compared to the PTZ group. It has been previously reported that administration of *C. zeylanicum* extract (200 and 400 mg/kg) for 21 days significantly reversed scopolamine-induced amnesia, reduced MDA level and increased GSH level in the brain tissues of rats (Jain et al., 2015 ▶). In an animal model of diabetes, *C. zeylanicum* extract (100, 200 and 400 mg/kg) for 14 days significantly reduced latency time and distance in Morris water maze and increased hippocampal cell density and activity of CAT and GPx enzymes in comparison with the STZ group (Edalatmanesh et al., 2018 ▶), which support the preventive effect of this plant on dysregulation of oxidant and antioxidant biomarkers. Considering the results of the present study, it seems that medium and high doses (200 and 400 mg/ kg) of the extract had better protective effects than the low dose (100 mg/kg) on oxidative stress. It was also observed that only 400 mg/kg of the extract improved thiol content in the hippocampus and cortex but the medium and high doses had no significant effect.

The GC-MS analysis of essential oil of *C. zeylanicum* has suggested that the main compounds present in this extract were (E)-cinnamaldehyde, linalool, ß-caryophyllene, eucalyptol and eugenol (Alizadeh Behbahani et al., 2020 ▶). Since linalool is one of the main constituents of *C. Zeylanicum*, the GABAergic system modulation by linalool was proposed. The data suggest that the anticonvulsant mode of action of linalool includes a direct interaction with the NMDA receptor complex and GABA (A) receptors (Silva Brum et al., 2001 ▶).

Therefore, the antiepileptic effect of *C. zeylanicum* extract is likely to be exerted also in the PTZ-induced seizure model to increase cerebral GABA content. Linalool is a monoterpene which is present in many aromatic oil essences as a main component and could be responsible for antiepileptic effect of *C. zeylanicum*.

Concentration-dependent effects of the extract of *C. zeylanicum* on all measured variables were observed in the present study. The effect of the two higher doses was higher than the low dose. In addition, the effect of high concentration of *C. zeylanicum* was also higher than the medium dose. 

Current results reveal that *C. zeylanicum* hydroethanolic extract has anticonvulsant actions. This behavior in brain tissues is followed by an antioxidant effect. Additional studies are needed to investigate the anticonvulsant molecular mechanisms and phytochemical studies are suggested to be done to characterize and isolate the components responsible for the anticonvulsant activity of* C. zeylanicum*.

## Conflicts of interest

The authors have declared that there is no conflict of interest.
